# In Vitro Comparison of Dye Penetration through Four Temporary Restorative Materials

**Published:** 2010-05-20

**Authors:** Shahriar Shahi, Mohammad Samiei, Saeed Rahimi, Hossein Nezami

**Affiliations:** 1. Department of Endodontics, Dental School, Tabriz University of Medical Sciences, Tabriz, Iran.; 2. Department of Endodontics, Dental School, Tabriz University of Medical Sciences, Tabriz, and Iranian Center for Endodontic Research, Tehran, Iran.; 3. Endodontist, Tabriz, Iran.

**Keywords:** Coronal Microleakage, Dye Penetration, Temporary Fillings

## Abstract

**INTRODUCTION:**

The purpose of this in vitro study was to compare the coronal seal of four temporary filling materials, Coltosol, Zonalin, Zamherir, and Intermediate Restorative Material (IRM) by the India ink dye penetration test.

**MATERIALS AND METHODS:**

Endodontic access preparations were prepared in 120 extracted intact human premolars. The teeth were randomly divided into six groups including four experimental and two control groups. The access cavities in each group were sealed with Coltosol, Zonalin, Zamherir, and IRM; subsequently thermocycling was applied for 5-55˚C for 150 cycles. The teeth were immersed in 10% India ink for 72 hours to assess leakage. The teeth were then rinsed, dried, and sectioned mesiodistally and evaluated under a stereomicroscope for dye penetration. Data were analyzed using one-way ANOVA and post hoc Tukey tests.

**RESULTS:**

Positive control specimens showed complete dye penetration, while negative controls had no penetration. In the experimental groups, the lowest and highest leakage scores were observed in the Zonalin and Coltosol groups, respectively (P<0.05). There were no statistically significant differences in marginal leakage between Zonalin-Zamherir and Coltosol-IRM groups.

**CONCLUSION:**

These results suggest that Zonalin and Zamherir have a superior seal and less micro-leakage into the canals compared to the two other materials.

## INTRODUCTION

An appropriate temporary filling material can be an important factor which determines the success or failure of root canal treatment. These materials seal the tooth temporarily, preventing the entry of fluids, microorganisms, and other debris into the root canal space. In addition, they prevent the escape of medicaments which were placed in the pulp chamber and root canal system [1][2][3][4][5].

A coronal filling material is considered to be effective when it is able to fulfill certain properties including good sealability, dimen-sional stability, abrasion and compression resistance, lack of porosity, easy handling, compatibility with intracanal medicaments, and good esthetic appearance [6].

There are a whole host of types of temporary filling materials available to the clinician, each with different compositions, setting mechanisms, and microstructures. Coltosol is a pre-manipulated eugenol-free material which sets in contact with moisture; however it has demonstrated conflicting results when its marginal seal was assessed [7][8][9]. Lee et al. noted that the effective sealing ability of Cavit could be due to its expansion during setting which is related to its hygroscopic properties. However, Cavit is not esthetic and it does not resist masticatory loads [10].

Intermediate Restorative Material (IRM), a zinc oxide-eugenol (ZOE) based material, has been associated with antibacterial activity [7]. Together with Cavit, IRM has been the most commonly used temporary filling material in endodontics, even though its sealing capability has demonstrated conflicting results [11][12][13].

Zaia et al. evaluated the microleakage of Coltosol, IRM, Vidrion R and Scotch Bond temporary restorative materials using dye penetration. All the four restorations showed dye penetration. IRM and Coltosol produced the best seal; and Scotch Bond had the highest microleakage [8]. Zmener et al. evaluated the microleakage of IRM, Cavit, and UltraTemp Firm. All the specimens showed microleakage at the restoration-dentin interface whilst IRM showed additional bulk microleakage [11].

Recently, a new Iranian ZOE-based material (Zamherir) has been introduced as a temporary restorative material with a composition similar to Zonalin. The purpose of the this in vitro study was to compare the sealing ability of temporary filling materials including zinc oxide-calcium sulfate (Coltosol), zinc oxide-eugenol (IRM) and Zonalin with a new temporary material (Zamherir) using India ink dye penetration test.

## MATERIALS AND METHODS

One hundred and twenty extracted, intact, and caries-free human premolars were selected for this study. These teeth were immersed in NaOCl 5.25% (Pakshoma, Tehran, Iran) for 5 minutes to disinfect teeth and remove the soft tissue from the root surfaces. Subsequently, teeth were rinsed and stored in normal saline. The same operator prepared standardized access cavities.

Working lengths were determined using K-file size #15 (Dentsply Maillefer, Ballaigues, Switzerland) 0.5 mm short of the apex. Root canal cleaning and shaping was carried out using the step-back technique. Initially, K-files #15-35 were used to prepare the apical third of the root canals. Gates Glidden burs #2 to #4 (Mani, Japan) were used to prepare the middle and coronal thirds of the root canals. Approximately 2 mL of sodium hypochlorite solution 2.5% was used for irrigation between each instrumentation procedure. After cleaning and shaping, the root canals were dried with paper points and obturated with cold laterally condensed gutta-percha (Ariadent, Tehran, Iran) and ZOE sealer. When root canals’ obturations were completed, a hot instrument and a plugger were used to remove excessive gutta-percha and to ensure good condensation in the coronal part of the root obturation. In this way, a minimum of 4-6 mm coronal space was available for the temporary restorative material.

The teeth were randomly divided into 6 groups (4 experimental and 2 control groups) of 20 premolar teeth each. The teeth in the positive controls were not filled with restorative materials; only a small dry cotton pellet was placed in the pulp chamber. In the negative control group cavities were completely filled with inlay wax (Kerr, Oklahoma, USA). In the four experimental groups, all the materials were mixed according to manufacturer’s instructions by the same operator. After placement of the test materials [Coltosol (Coltene, Altstatten, Switzerland), Zonalin (Purton, Wiltshin, Sweden), Zamherir (Ajdarou, Ardebil, Iran), and IRM (zinc oxide-eugenol; Dentsply; Milford, DE, USA powder-to-liquid ratio of 6 g/mL)] into the access cavities, the specimens were stored in an incubator at 37˚C at 100% humidity for 24 hours. The specimens were thermocycled for 500 cycles in distilled water at 5-55˚C; i.e. 30 seconds in each bath. After thermocycling, the specimens were air dried. The teeth in the negative control group were completely covered with two layers of nail varnish. The experimental groups and positive control group were also coated twice except the occlusal surfaces.

All the specimens were placed in India ink 10% (AB Chemi, Glasco, UK) at 37˚C and 100% humidity for 72 hours (3 days). Subsequently, they were removed from the dye solution, irrigated under tap water, and air dried.

The specimens were sectioned into two parts along their longitudinal axis in a mesiodistal direction with a diamond disc (D&Z, Munchen, Germany). The specimens were viewed and photographed using a stereomicroscope (Olympus SZ, 9-ILL B200-Chiyoda KU, Japan) with ×10 magnifications. The greatest depth of dye penetration along the wall of the access cavity and the root of both tooth segments was selected and recorded. Measurements of dye penetration were carried out by same operator. Results were analyzed using one-way ANOVA and post hoc Tukey tests (P<0.05).

## RESULTS

**Figure 1:**
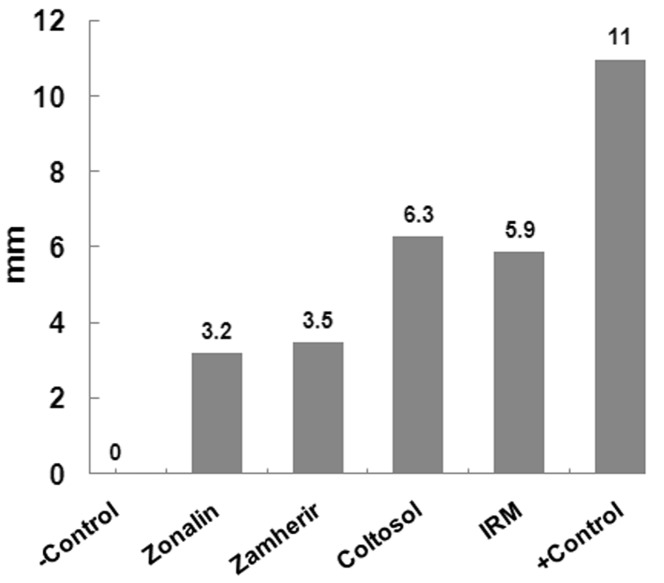
Mean marginal leakage observed for the different temporary filling materials and control groups.

The negative control group showed no dye penetration and the positive control group demonstrated maximum dye penetration. The mean marginal dye leakage scores for each group are presented in Figure 1. In the experimental groups, the lowest and highest leakage scores were observed in the Zonalin and Coltosol groups, respectively. There was significant difference between these two groups (P<0.05). The differences in marginal leakage between Zonalin and Zamherir and also between Coltosol and IRM groups were not significant.

## DISCUSSION

There is strong evidence that suggests that good post-obturation coronal seal can improve the prognosis of root-filled teeth [14][15]. some studies suggest that gutta-percha cannot prevent the passage of saliva nor the penetration of bacteria into the root canal and periapical tissues [5][11].

Providing a proper marginal seal with temporary filling material is necessary to minimize contamination of the root canal systems during and after root canal therapy. Temporary restorative materials should be used which reduce the leakage of saliva and microorganisms [7][10].

Coltosol is hygroscopic cement which expands twice as much as zinc oxide-eugenol when in contact with moisture (linear expansion); this is due to water sorption. This expansion provides good adaptation between the restorative material and cavity walls [8][16][17][18]; however, some authors believe that expansion of hygroscopic restorative materials leads to poor adaptation at the interface of restorative material and cavity walls [1][19]. The findings of the present study provide further evidence for the poor sealing ability of Coltosol.

IRM, is a zinc oxide-eugenol reinforced cement which, unlike Coltosol, requires mixing of its separate powder and liquid components before use [6][7]. In this study, the sealing ability of IRM was poor and showed significantly higher marginal leakage than the other temporary cements, confirming previous reports [4][9][10][12][20][21][22]. Studies have shown that variations in volume resulting from contraction of the material and the unhomogeneous mixing process could partially explain the poor sealing results with this filling material [4].

In this study, extracted intact premolars were used and a thickness of 4-6 mm of restorative material was placed. It has been reported that a minimum of 3.5-4 mm of temporary restorative material is necessary to prevent microleakage [1][11].

Thermocycling procedures attempt to stimulate temperature changes that take place in-vivo [23]. The temperature range used in thermocycling (5˚C and 55˚C), corresponds to the extremes of temperatures experienced in the oral environment the present study used thermocycling to simulate intraoral conditions.

Evaluation of microleakage with India ink dye penetration is one of the most commonly used methods. This black dye has small particles [24] that can easily penetrate by simple diffusion; it also has negligible influence on the sealer of root canal obturation. Moreover, it is not absorbed by the hydroxyapatite crystals of dentin [24][25] and is frequently used in for microleakage studies [8][26][27].

In the present study, a new ZOE-based material (Zamherir) was compared with three commonly used temporary filling materials. All experimental groups demonstrated leakage between the material and the access cavity walls. Zonalin group showed the least marginal leakage among experimental groups, whereas the Coltosol and IRM specimens showed maximum marginal leakage along the material-tooth interface.

However, another study has shown that IRM and Coltosol produce the greatest seal while Scotch Bond had the greatest microleakage [8].

According to Balto’s study which assessed Cavit, IRM, and TempBond, Cavit had the least and TempBond showed the greatest microleakage [9].

Kazemi et al. dye penetration test showed that Cavit is a more appropriate temporary endodontic restoration compared with Tempit and IRM, as it has better marginal stability and permeability [12].

Zmener et al. evaluated microleakage of IRM, Cavit, and UltraTemp Firm using methylene blue dye solution 2% for 10 days. All specimens showed microleakage at the interface of restoration and dentin whilst IRM showed additional bulk microleakage [11].

In this study, all the experimental specimens showed dye penetration and thus microleakage. Zonalin demonstrated good coronal sealing ability which statistically did not differ from that demonstrated by Zamherir. Coltosol and IRM both showed significantly greater microleakage.

## CONCLUSION

The findings of this in vitro study suggests that Zonalin and Zamherir temporary restorative materials have low microleakage and canal contamination in comparison to Coltosol and IRM.
